# *Bacillus tropicus* YJ33 and *Medicago sativa* L. Synergistically Enhance Soil Aggregate Stability in Saline–Alkali Environments

**DOI:** 10.3390/microorganisms13061291

**Published:** 2025-05-31

**Authors:** Jingjing Li, Yajuan Che, Shiyang Chen, Mengge Liu, Mengmeng Diao, Chao Yang, Wenke Jia

**Affiliations:** 1 College of Grassland Science, Qingdao Agricultural University, Qingdao 266109, China; lijingjing@qau.edu.cn (J.L.); cyj2490390935@163.com (Y.C.); csy20242152009@163.com (S.C.); 17860747980@163.com (M.L.); mmengdiao@126.com (M.D.); 2 Shandong Key Laboratory for Germplasm Innovation of Saline-Alkaline Tolerant Grasses and Trees, Qingdao 266109, China

**Keywords:** *Medicago sativa* L., *B. tropicus* YJ33, GMD, stability of soil aggregates

## Abstract

Soil salinization represents a significant global environmental challenge, necessitating the urgent amelioration of saline–alkali lands. As a critical functional component of the soil system, soil aggregates play a pivotal role in enhancing soil structure and are essential for nutrient cycling and plant growth. However, the synergistic effects of plants and microorganisms on alterations in soil aggregate composition, stability, and nutrient content in saline–alkali soils remain inadequately understood. In this study, three saline soil gradients from the Yellow River Delta were analyzed: low saline soil (S1, 1.65 g/kg), medium saline soil (S2, 4.54 g/kg), and high saline soil (S3, 6.57 g/kg). For each gradient, four experimental treatments were established: (1) inoculation of *Bacillus tropicus* YJ33 alone (B), (2) planting of alfalfa alone (M), (3) combined alfalfa cultivation with *B. tropicus* YJ33 inoculation (MB), and (4) an unamended control (CK). These treatments were implemented in controlled laboratory pot experiments to evaluate the individual and synergistic impacts of alfalfa and *B. tropicus* YJ33 on saline soil aggregate stability and structural organization. Overall, *B. tropicus* YJ33 inoculation significantly promoted the growth and nutritional quality of alfalfa. B, M, and MB treatment increased the contents of total carbon (TC), total nitrogen (TN), and available phosphorus (AP) and promoted the activities of soil alkaline phosphatase (S-ALP) and soil urease (S-UE) in the soil. Simultaneously, these treatments resulted in a reduction in the proportion of micro-aggregates, an increase in the proportion of large and small aggregates, and significantly enhanced mean weight diameter (MWD) and geometric mean diameter (GMD), improving the stability of soil aggregates. Random forest analysis identified AP, *B. tropicus* YJ33, salinity, TC, and available nitrogen (AN) as key determinants of alfalfa biomass. Partial least squares (PLS) modeling further corroborated the role of *B. tropicus* YJ33 in enhancing soil nutrient content, improving aggregate stability, and increasing alfalfa yield. In conclusion, *B. tropicus* YJ33 was demonstrated to enhance the stability of soil aggregates and nutrient availability in saline–alkali soils, thereby significantly promoting the growth, yield, and nutritional quality of alfalfa.

## 1. Introduction

Soil salinization has become a major global environmental issue, affecting more than 100 countries and areas [1], with over 424 million hectares of surface soils (0–30 cm) and 833 million hectares of subsurface soils (30–100 cm) currently affected worldwide [2]. In China, saline–alkali soils account for 4.88% of total arable land area [3], with the Yellow River Delta alone accounting for nearly 180,000 hectares [4]. It is predicted that by 2050, the global population will exceed 9 billion, necessitating at least a 70% increase in food production to meet rising demand [5] and innovative strategies to rehabilitate marginal lands while avoiding ecosystem simplification [6]. c maintaining the stability of grain production, and strictly abiding by the national arable land red line [7,8,9].

Saline–alkali soils are typically characterized by poor physical structure, with capillary action facilitating the upward migration of salts to the surface soil and exacerbating soil salinity [10]. Additionally, these soils contain high concentrations of Na^+^. When Na^+^ concentrations become excessively high, these ions occupy a number of cation exchange sites on soil particle surfaces, displacing other cations and increasing repulsive forces between particles. This destabilizes the previously stable soil aggregate structure, diminishing its stability [11]. The resulting deterioration of soil structure degrades physical properties, reduces water retention, and disrupts nutrient cycling, all of which severely impact agricultural productivity. Thus, enhancing the aggregate structure of saline–alkali soils is a critical strategy for improving soil quality and promoting sustainable agricultural development.

Soil aggregates, the fundamental structural units of soil, are pivotal for enhancing soil structure [12]. Their formation and stability depend closely on soil organic carbon (SOC) and nutrient accumulation [13]. The size, quantity, proportion, and morphology of aggregates effectively indicate structural stability, directly influencing soil physical, chemical, and biological properties [14]. Research shows that aggregate particle size distribution is critical for characterizing soil structure. It also plays a key role in nutrient storage, soil aeration optimization, and SOC accumulation [15]. Soil aggregates are generally categorized into macro-aggregates (>0.25 mm) and micro-aggregates (<0.25 mm) based on particle size [16]. Macro-aggregates consist mainly of organic residues, plant roots, and fungal hyphae, along with other adhered materials [17], while micro-aggregates form through inorganic colloids (clay/silt) bound by microbial or root-derived polysaccharides [18]. Compared to micro-aggregates, macro-aggregates exhibit better breathability and contain abundant fresh organic matter, providing material and energy resources for soil microorganisms [19]. Therefore, the primary objective of saline–alkali land reclamation is to reduce salinity, lower pH, increase the content of macro-aggregates, and improve soil structure.

In recent years, a significant amount of research has been conducted on improving the aggregation and stability of saline–alkali soils. The vast majority of research has focused on soil organic amendments [20], straw returning to the field [21], farming system [22], organic fertilizer application [23,24,25], land-use change [5,26], and biochar addition [27,28,29] on aggregate stability. These interventions have significantly improved soil structure and promoted the formation of macro-aggregates. However, there is still relatively little research on enhancing the aggregate stability of saline–alkali soils through the cultivation of salt-tolerant plants and the inoculation of plant growth-promoting rhizobacteria (PGPR). The cultivation of salt-tolerant plants not only enhances the stability of soil aggregates but also significantly increases soil nutrients [30,31] and augments the diversity and abundance of microorganisms in saline–alkali soils [32]. The synergistic interaction between salt-tolerant plants and rhizosphere microorganisms presents a novel approach to reclaiming saline–alkali soils, thereby enhancing long-term soil productivity and ecological stability.

Alfalfa (*Medicago sativa* L.), a perennial leguminous forage crop, exhibits extensive root architecture, vigorous regrowth potential, and high nutritional value, as well as its demonstrated resilience to saline–alkali stress [33]. The cultivation of alfalfa significantly enhances soil fertility, promotes vegetation restoration in degraded ecosystems, and sustains agricultural productivity. Furthermore, alfalfa cultivation provides critical ecosystem services, including soil erosion mitigation, prevention of secondary salinization–alkalization in agricultural soils, and enhancement of soil structural. Rhizobia colonizing the alfalfa rhizosphere function as PGPR, whose secreted exopolysaccharides (EPSs) enhance soil aggregation by binding soil particles [34]. *B. tropicus* YJ33, a recognized PGPR, enhances root system development and promotes root physical penetration and entanglement of roots with the soil. This process improves soil aggregate stabilization. Furthermore, EPS produced by *B. tropicus* act as binding agents, effectively stabilizing soil particle cohesion and reinforcing aggregate stability [35]. While salt-tolerant vegetation and PGPR have been shown to enhance soil structural properties, their synergistic interactions in mediating soil aggregate formation and stabilization remain poorly characterized. Consequently, further research is warranted to elucidate this mechanism.

The objectives of this study are delineated as follows: (i) to examine the effects of *B. tropicus* YJ33 inoculation on the growth and nutritional quality of alfalfa across varying alkalinity gradients; (ii) to investigate the synergistic interactions between alfalfa cultivation and *B. tropicus* YJ33 inoculation on soil physicochemical properties and enzymatic activities; and (iii) to assess the enhancement of soil aggregate stability under different salinity and alkalinity gradients as a result of alfalfa cultivation and *B. tropicus* YJ33 inoculation. This study seeks to provide a comprehensive understanding of the combined impact of alfalfa and *B. tropicus* YJ33 on improving soil structure and plant growth in saline–alkaline environments, with a particular emphasis on augmenting soil quality and stability. Through this research, we aim to explore the potential application of this dual approach in sustainable land management and soil remediation. We hypothesized that the symbiotic relationship between *B. tropicus* YJ33 and alfalfa has the potential to enhance the stability of soil aggregates. This synergistic interaction is likely to manifest through increased microbial activity, improved rhizosphere conditions, and augmented soil nutrient levels.

## 2. Materials and Methods

### 2.1. Experimental Design

In this study, low saline soil (S1, 1.65 g/kg), medium saline soil (S2, 4.54 g/kg), and high saline soil (S3, 6.57 g/kg) were collected from the Yellow River Delta Agricultural High-Tech Industry Demonstration Zone in Dongying City, Shandong Province, China (37.3° N, 118.7° E; altitude 6.7 m). The area has a warm temperate continental monsoon climate, with an average annual temperature of 12.8 °C and an average annual precipitation of 555.9 mm. For each gradient, four experimental treatments were established: (1) inoculation of *B. tropicus* YJ33 alone (B), (2) planting of alfalfa alone (M), (3) combined alfalfa cultivation with *B. tropicus* YJ33 inoculation (MB), and (4) an unamended control (CK). Plastic pots (size: 26 × 26 cm) were filled with 8 kg of soil for the pot culture experiment. Each treatment has 4 pots, totaling 48 pots (3 × 4 × 4). These treatments were implemented in controlled laboratory pot experiments to evaluate the individual and synergistic impacts of alfalfa and *B. tropicus* YJ33 on saline soil aggregate stability and structural organization.

The alfalfa variety utilized in this study was ‘Zhong mu No. 3’, sourced from Beijing Best Grass Seed Co., Ltd. (Beijing, China), exhibiting a seed purity of 98% and a germination rate of 85%. Alfalfa seeds were germinated in a light incubator on 18 June 2023, under controlled conditions comprising a photoperiod of 16 h of light and 8 h of darkness, with daytime and nighttime temperatures set at 28 °C and 22 °C, respectively, and humidity levels maintained between 50% and 80%. Upon full leaf development on 22 June 2023, uniform seedlings were selected for transplantation, with 30 seedlings being transplanted into each pot. Following the emergence of the first true leaf, the seedlings were thinned, resulting in 15 alfalfa plants remaining in each pot. The greenhouse conditions were maintained with a photoperiod of 16 h of light and 8 h of darkness, with humidity levels controlled between 50% and 60%.

The strain used in this experiment was *B. tropicus* YJ33, previously isolated and characterized by our research group from the rhizosphere of *Suaeda salsa*. Strain identification was performed through 16S rRNA gene sequencing analysis. Previous investigations by our group have shown that this strain belongs to the salt-tolerant bacterium and is able to increase the salt-tolerance of alfalfa. Functional characterization revealed its dual capabilities in phosphate solubilization and indole-3-acetic acid (IAA) biosynthesis. The phylogenetic tree comparison of this strain is shown in our previous study [36].

The strain was inoculated into a beef extract peptone liquid medium under sterile conditions within a laminar flow hood and cultured at 28 °C with agitation at 180 r/min for 24 h. Following incubation, the culture was centrifuged at 4 °C at 4000 r/min for 5 min, and the supernatant was discarded. The bacterial suspension was subsequently resuspended in sterile water to create an inoculum with an optical density (OD_600_) of 2.0 ± 0.01 for subsequent use. During the experimental procedure, 300 mL of the bacterial inoculum was applied to each pot of soil monthly, resulting in a total of five applications. Both the control group and the pure alfalfa treatment group were provided with an equivalent volume of sterile water. To mitigate potential experimental errors stemming from variations in light exposure, the positions of the pots were systematically adjusted on a weekly basis.

### 2.2. Collection of Plant and Soil Samples

The three harvests of alfalfa took place on 30 August 2023, 12 November 2023, and 24 December 2023. During the initial flowering stage of alfalfa, plant height was measured in its natural state. The harvested material was placed in envelopes and subjected to an initial drying process at 105 °C for 30 min to arrest enzymatic activity and halt metabolic processes, thereby preventing sample degradation. Subsequently, the samples were transferred to a drying oven set at 65 °C, where they remained until a constant weight was achieved, ensuring complete moisture removal. The dried samples were then weighed using a high-precision electronic balance. The dried alfalfa was finely ground for subsequent chemical analyses.

In addition to the collection of plant samples, data pertaining to root characteristics, specifically root length and root biomass, as well as soil samples, were obtained following the third harvest of alfalfa. The plant roots were carefully extracted from the pots by gently tapping the sides or softly shaking the pots to facilitate the loosening of soil surrounding the roots. The roots were then rinsed with water in a cautious manner to avoid excessive washing that might cause damage. Following the washing process, root length was measured. Subsequently, the roots were placed in an envelope and subjected to an initial drying phase at 105 °C for 30 min. Thereafter, the samples were transferred to a drying oven set at 65 °C, where they were maintained until a constant weight was achieved, ensuring the complete removal of moisture. A five-point sampling method was utilized to gather soil samples from two distinct depth layers: 0–10 cm and 10–20 cm. The five soil cores from each depth were amalgamated to create a composite sample for the respective layer. The collected soil samples underwent measurements of aggregate stability, nutrient content analysis, and assays for soil enzyme activity.

### 2.3. Soil Aggregate Fractionation

All soil samples were manually collected from the alfalfa root zone and gently fragmented along the natural soil surface cracks to facilitate the analysis of soil aggregate composition. A 500 g soil sample was sequentially dry-sieved through 2 mm and 0.25 mm mesh screens to separate three aggregate size fractions: large macro-aggregates (>2 mm), small macro-aggregates (0.25–2 mm), and micro-aggregates (<0.25 mm). Then, these aggregates were divided into two subsamples for distinct analytical workflows: one for physicochemical characterization and enzyme activity measurements and the other for subsequent wet-sieving procedures.

Soil aggregate stability was quantitatively assessed using a standardized wet-sieving protocol implemented on a TPF-100 Soil Aggregate Analyzer (Zhejiang Top Cloud-agri Technology Co., Ltd., Hangzhou, China). A composite sample (50 g) was reconstituted proportionally from dry-sieved aggregate fractions to preserve original size distribution. The analysis involved four sequential phases: (1) immerse the nested sieves (2 and 0.25 mm) along with the soil samples in deionized water for 10 min, ensuring that the aggregates are fully saturated and absorb water; (2) the experimental setup was initiated to perform vertical oscillation of the sieves in distilled water, with an amplitude of 5 cm, for a duration of 30 min; (3) collect the samples retained by each sieve layer by layer and transfer them to pre-weighed aluminum containers (dry at 105 ± 2 °C until constant weight is achieved); (4) place the aluminum containers in an oven at 105 ± 2 °C for drying for 6 h, then cool for 30 min and weigh. The soil water-stable aggregate content is calculated using the following formula:

(1) $${WSA}_{i}\left(\%\right)=\frac{m_{i}}{M_{total}}\times 100$$

In the formula, *WSA_i_* represents the mass percentage of water-stable aggregates of the *i*-th particle size (%), $m_{i}$ is the dried mass of the sample retained by the *i*-th sieve (g), and $M_{total}$ is the total dried mass of all particle sizes (g).

In addition, the mean weight diameter (MWD) and mean geometric diameter (GMD) of soil aggregates after wet sieving were calculated. MWD reflects the stability of the soil structure, with larger values indicating a high proportion of larger aggregates, a more stable soil structure, and a greater resistance to erosion (e.g., wind and water erosion). GMD is more sensitive to the proportion of small- and medium-sized aggregates, which can reveal the homogeneity of the soil texture. Smaller GMD may indicate more micro-agglomerates. Both can be used to quantitatively evaluate the stability of soil aggregates. Their calculation formulas are as follows:

(2) $$MWD=\sum_{i=1}^{n}x_{i}w_{i}$$

(3) $$GMD=\frac{\sum_{i=1}^{n}w_{i}\times \ln x_{i}}{\sum_{i=1}^{n}w_{i}}$$

In the formula, $w_{i}$ represents the proportion of aggregates in different soil particle size fractions, and $x_{i}$ represents the average diameter of the soil particles in each fraction.

### 2.4. Determination of Relevant Indexes of Plant and Soil Samples

Determination of crude protein (CP) content of alfalfa: alfalfa: samples were initially sieved through a 60-mesh screen. Subsequently, 10 mg of the sieved sample was precisely weighed and placed in a tin foil boat. The total nitrogen content of the sample was then determined using an elemental analyzer (Vario EL cube, Elementar Analysensysteme GmbH, Langenselbold, Germany). The CP content was subsequently calculated from the measured total nitrogen content by applying a conventional conversion factor of 6.25.

Determination of total phosphorus (TP) content of alfalfa: the ground alfalfa samples underwent acid digestion using the H_2_SO_4_-H_2_O_2_ method. Following digestion, the TP content of alfalfa was quantitatively analyzed using a continuous flow analyzer (AutoAnalyzer3, SEAL Analytical, Norderstedt, Germany).

Determination of neutral detergent fiber (NDF) and acid detergent fiber (ADF) content of alfalfa: initially, a 0.5 g plant sample was accurately weighed, and the NDF content was quantified utilizing a fiber analyzer. Following the NDF assessment, the sample was subjected to drying and subsequent weighing. The same plant sample, post-NDF determination, was then employed to ascertain the ADF content.

Determination of total carbon (TC) and total nitrogen (TN) content in the soil: a precisely measured 20 mg soil sample was weighed using a tin foil boat (Elementar Analysensysteme GmbH, Langenselbold, Germany). The samples were subsequently analyzed with an elemental analyzer (Vario EL cube, Elementar Analysensysteme GmbH, Langenselbold, Germany), facilitating the simultaneous quantification of TC and TN content in the soil samples.

Determination of available nitrogen (AN) content in the soil: AN was extracted from the soil using a 2 mol/L KCl solution. Following extraction, the AN content in the soil samples was analyzed using a continuous flow analyzer (AutoAnalyzer3, SEAL Analytical, Norderstedt, Germany).

Determination of available phosphorus (AP) content in the soil: phosphorus was extracted from the soil using a 0.5 mol/L NaHCO_3_ solution. Following extraction, the AP content in the soil samples was analyzed using a continuous flow analyzer.

### 2.5. Determination of Soil Enzyme Activity

In this study, we quantified the activity of two soil hydrolytic enzymes: soil urease (S-UE) and soil alkaline phosphatase (S-ALP), both of which play crucial roles in the acquisition of nitrogen and phosphorus in soil ecosystems. The activities of these enzymes were determined using a microplate reader (Magellan Tracker 7.3, Tecan Austria GmbH, Grödig, Austria).

Determination of S-UE activity: the indophenol blue colorimetric method was used. The kits were purchased from Suzhou Keming Biotechnology Co., Ltd. (Suzhou, China), and the absorbance values were measured at a wavelength of 578 nm, as described in the instruction manual.

Determination of S-ALP activity: the activity of S-ALP was calculated by catalyzing the hydrolysis of disodium phosphate to produce phenol and sodium dihydrogen phosphate, and the amount of phenol produced was measured. The kits were purchased from Suzhou Keming Biotechnology Co., Ltd., and the absorbance values were measured at 660 nm, as described in the instruction manual.

### 2.6. Statistical Analysis

Data analysis was conducted using Microsoft Excel 2019 and SPSS 27.0 for data organization and processing. To evaluate significant differences among treatments, a one-way analysis of variance (ANOVA) was employed, followed by the Least Significant Difference (LSD) method for multiple comparisons. All data are reported as mean ± standard error to accurately convey central tendency and variability. Furthermore, bar plots and box plots were generated using Origin 2024 software to visually represent data distribution and treatment differences. Random forest prediction and partial least squares (PLS) structural equation modeling (SEM) were performed using R (version 4.4.2) to investigate the primary factors affecting alfalfa biomass and to analyze the interactions among soil physicochemical properties, soil aggregates, and crop yield.

## 3. Results

### 3.1. The Growth-Promoting Effect of B. tropicus YJ33 on Alfalfa Under Different Saline–Alkaline Gradients

**Figure 1:**
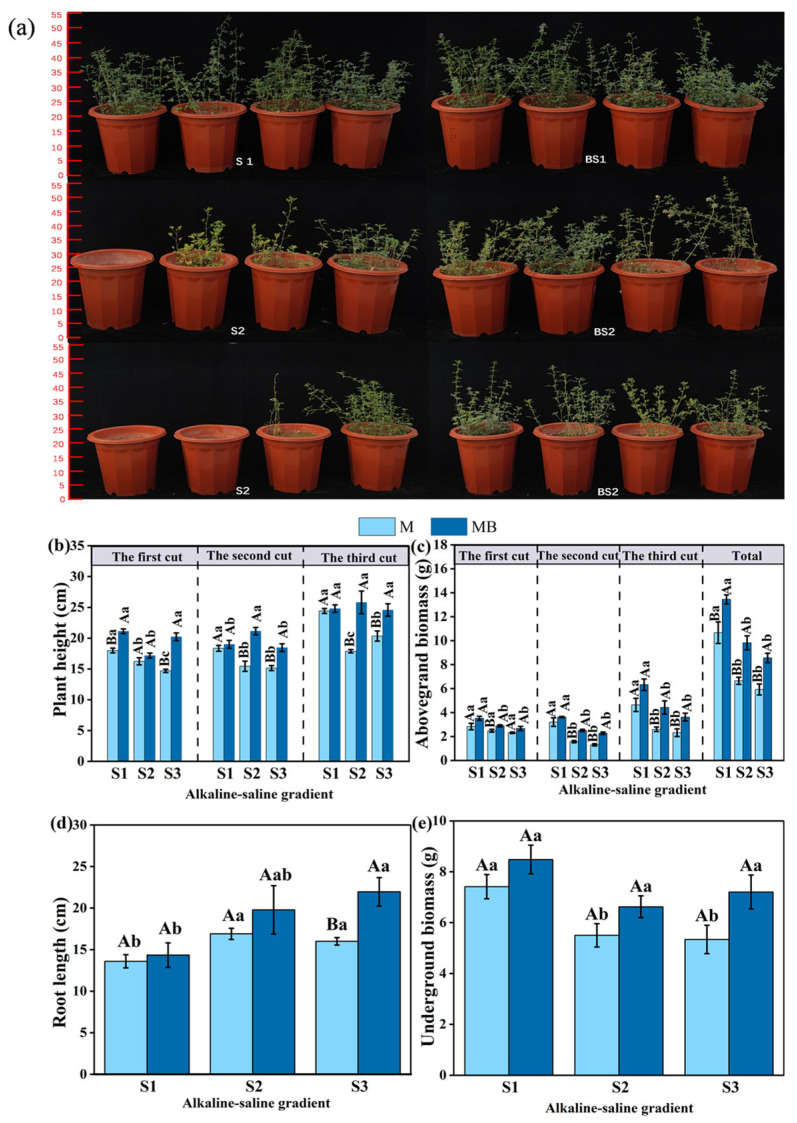
The impact of *B. tropicus* YJ33 inoculation on growth status (**a**), plant height (**b**), above ground biomass (**c**), root length (**d**) and underground biomass (**e**) of alfalfa. Lowercase letters show significant differences between salinity–alkalinity gradients (*p* < 0.05), and uppercase letters show significant differences between inoculated and non-inoculated treatments (*p* < 0.05). Values are expressed as mean ± SE (*n* = 4). Abbreviations: S1: low saline soil, S2: medium saline soil, S3: high saline soil, M: planting of alfalfa alone, and MB: combined alfalfa cultivation with *B. tropicus* YJ33 inoculation.

The growth performance of alfalfa inoculated with *B. tropicus* YJ33 significantly surpassed that of non-inoculated plants (Figure 1a). The MB treatment exerted a significant influence on the agronomic traits of alfalfa. Under varying salinity–alkalinity gradients, the plant height of alfalfa subjected to the MB treatment was significantly greater than that observed in the M treatment (*p* < 0.05), thereby enhancing alfalfa growth (Figure 1b). Across different salinity–alkalinity conditions, the aboveground biomass of alfalfa in the MB treatment consistently exceeded that in the M treatment (Figure 1c). As salinity–alkalinity levels increased, a decreasing trend in aboveground biomass was observed for alfalfa in both the MB and M treatments (Figure 1c).

Relative to the M treatment, the total aboveground biomass of alfalfa in the MB treatment increased significantly by 26.14%, 47.78%, and 44.53% across increasing salinity–alkalinity gradients (Figure 1c). This suggests that *B. tropicus* YJ33 exhibits strong growth-promoting effects under elevated salinity–alkalinity conditions. With rising salinity–alkalinity, the total aboveground biomass in both MB and M treatments showed a declining trend. Under the S2 and S3 salt gradient, the total aboveground biomass was significantly reduced compared to that under S1 salt gradient (*p* < 0.05) (Figure 1c).

Notably, root length and underground biomass were greater in the MB treatment than in the M treatment, though not statistically significant (*p* > 0.05) (Figure 1d,e). With increasing salinity–alkalinity, root length in both treatments increased, peaking at 21.96 cm (Figure 1d), whereas underground biomass decreased (Figure 1e). These findings suggest that while salinity–alkalinity stress may induce adaptive root growth, it concurrently suppresses overall biomass accumulation.

### 3.2. Effects of B. tropicus YJ33 on the Nutritional Quality of Alfalfa Under Different Saline–Alkaline Gradients

**Figure 2:**
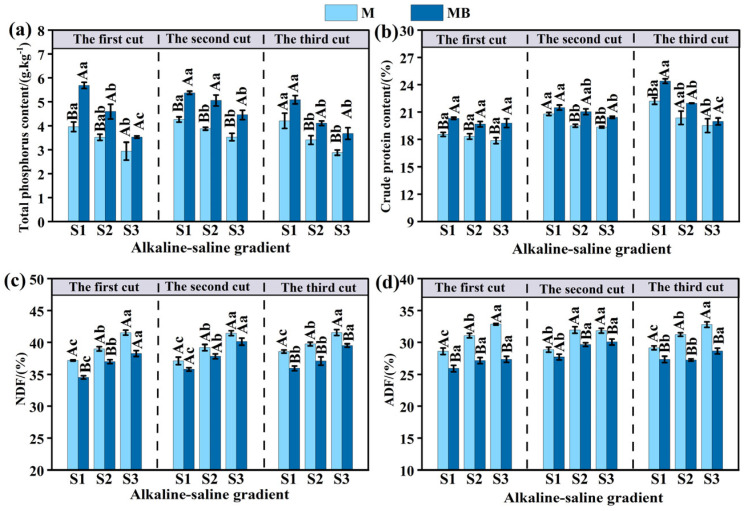
The impact of *B. tropicus* YJ33 inoculation on the total phosphorus (**a**), crude protein (**b**), NDF (**c**), and ADF (**d**) content in alfalfa. Lowercase letters show significant differences between salinity–alkalinity gradients (*p* < 0.05), and uppercase letters show significant differences between inoculated and non-inoculated treatments (*p* < 0.05). Values are expressed as mean ± SE (*n* = 4). Abbreviations: S1: low saline soil, S2: medium saline soil, S3: high saline soil, NDF: neutral detergent fiber, ADF: acid detergent fiber, M: planting of alfalfa alone, and MB: combined alfalfa cultivation with *B. tropicus* YJ33 inoculation.

The total phosphorus content in alfalfa subjected to the MB treatment was significantly greater than that observed under the M treatment (*p* < 0.05). For both the M and MB treatments, a declining trend in total phosphorus content was evident with increasing salinity–alkalinity levels. Notably, under the S3 salt gradient, the total phosphorus content in alfalfa was significantly lower compared to the S1 salt gradient (Figure 2a) (*p* < 0.05). Similarly, the crude protein content in alfalfa was significantly elevated under the MB treatment relative to the M treatment (*p* < 0.05), and both treatments exhibited a decrease in crude protein content with rising salinity–alkalinity gradients. This trend was not significant during the first harvest but became significant in the second and third harvests (Figure 2b). Furthermore, the MB treatment significantly reduced the NDF and ADF content in alfalfa compared to the M treatment. Both MB and M treatments demonstrated an increasing trend in NDF and ADF content with higher salinity–alkalinity gradients (Figure 2c,d). Under the S3 salt gradient, the contents of NDF and ADF in alfalfa were significantly greater than those observed under the S1 salinity–alkalinity gradient (Figure 2c,d) (*p* < 0.05).

### 3.3. Effects of Planting Alfalfa and Inoculating B. tropicus YJ33 on Soil Nutrients

**Figure 3:**
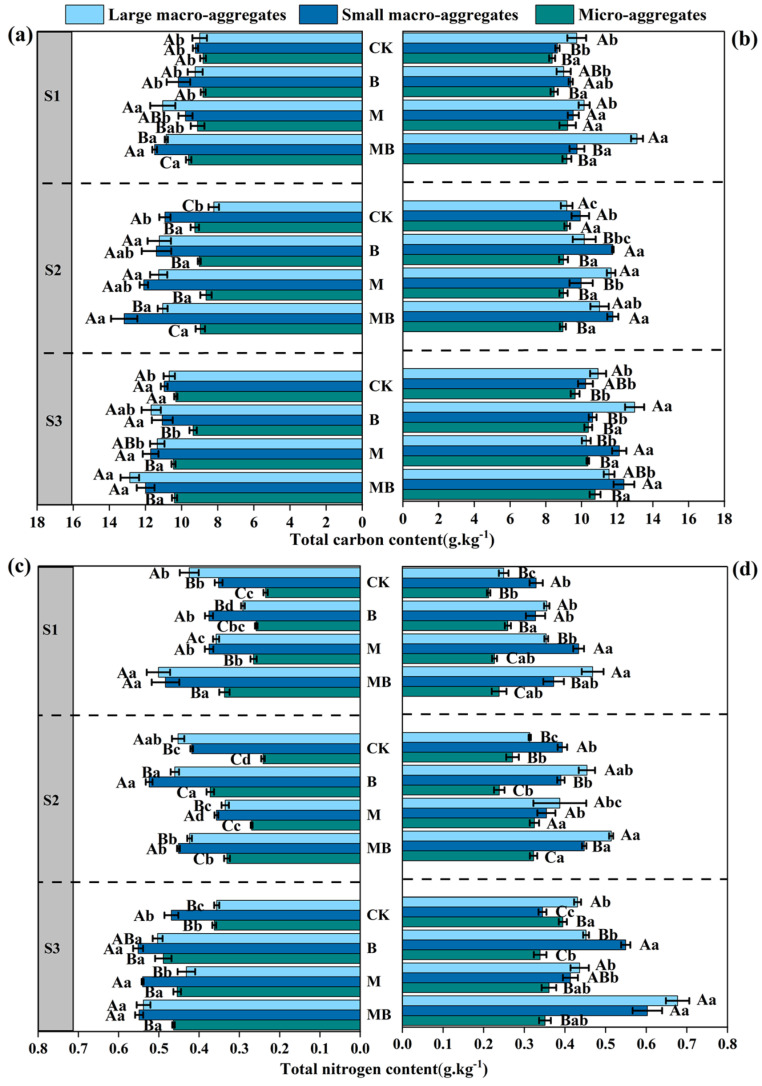
Soil total carbon (**a**,**b**) and total nitrogen (**c**,**d**) contents in aggregates affected by different treatments. Measurements for (**a**,**c**) pertain to the 0–10 cm soil layer, while (**b**,**d**) correspond to the 10–20 cm soil layer. Lowercase letters show significant differences in soil nutrient content among different treatments within the same salinity–alkalinity gradient (*p* < 0.05), and uppercase letters show significant differences in soil nutrient content across different soil particle sizes (*p* < 0.05). Values are presented as mean ± SE (*n* = 4). Abbreviations: S1: low saline soil, S2: medium saline soil, S3: high saline soil, CK: unamended control, B: inoculation of *B. tropicus* YJ33 alone, M: planting of alfalfa alone, and MB: combined alfalfa cultivation with *B. tropicus* YJ33 inoculation.

As salinity–alkalinity levels increased, the TC content in the soil exhibited a declining trend (Figure 3a,b). Across all salinity–alkalinity gradients, the B and M treatments generally demonstrated higher TC content compared to the CK treatment, with the MB treatment displaying the highest TC content, particularly under the S1 and S2 salt gradients. Conversely, large macro-aggregates consistently exhibited higher TC content, suggesting a superior capacity for organic carbon retention, whereas small macro-aggregates and micro-aggregates typically showed lower TC content (Figure 3a,b).

Additionally, as salinity–alkalinity increased, the TN content in the soil also displayed a decreasing trend (Figure 3c,d). Across all salinity–alkalinity gradients, both the B and M treatments demonstrated higher TN content compared to CK treatment, particularly within the 0–10 cm soil layer. Notably, the MB treatment exhibited greater TN content than either the B or M treatments alone (Figure 3c,d). This suggests that the synergistic interaction of these treatments not only enhances nitrogen fixation and transformation but also augments nitrogen retention in the soil through increased organic matter and microbial activity. Additionally, large macro-aggregates were found to possess higher total nitrogen content than micro-aggregates, indicating a superior nitrogen retention capacity.

**Figure 4:**
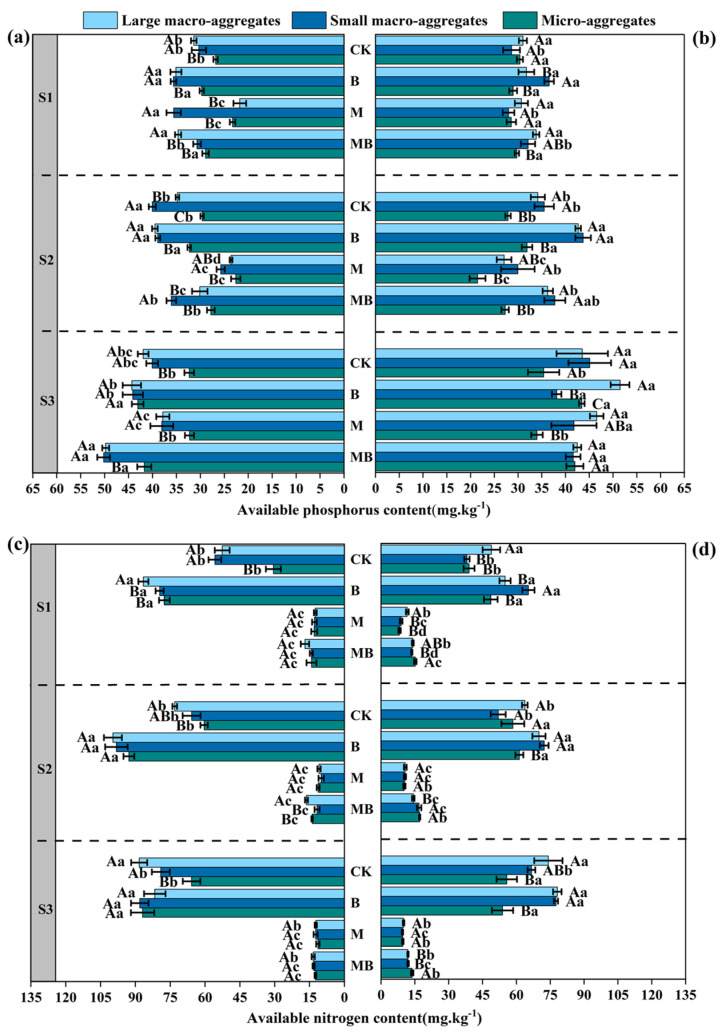
Soil available phosphorus (**a**,**b**) and available nitrogen (**c**,**d**) contents in aggregates affected by different treatments. Measurements for (**a**,**c**) pertain to the 0–10 cm soil layer, while (**b**,**d**) correspond to the 10–20 cm soil layer. Lowercase letters show significant differences in soil nutrient content among different treatments within the same salinity–alkalinity gradient (*p* < 0.05), and uppercase letters show significant differences in soil nutrient content across different soil particle sizes (*p* < 0.05). Values are presented as mean ± SE (*n* = 4). Abbreviations: S1: low saline soil; S2: medium saline soil; S3: high saline soil, CK: unamended control, B: inoculation of *B. tropicus* YJ33 alone, M: planting of alfalfa alone, and MB: combined alfalfa cultivation with *B. tropicus* YJ33 inoculation.

As salinity–alkalinity levels increased, the AP content in the soil exhibited a declining trend (Figure 4a,b). Under all salinity–alkalinity gradients, the B treatment generally displayed higher AP content, particularly under the S1 salt gradient. *B. tropicus* YJ33 appears to enhance phosphorus availability in the soil by facilitating phosphorus dissolution and transformation. In contrast, the AP content in the M treatment decreased relative to the CK, while the AP content in the MB treatment increased compared to the M treatment (Figure 4a,b). The observed effects of the MB treatment are likely attributable to the synergistic interaction between alfalfa and *B. tropicus* YJ33. Across all salinity–alkalinity gradients, it was observed that large macro-aggregates generally exhibited higher available phosphorus (AP) content, suggesting a greater capacity for AP retention compared to small macro-aggregates and micro-aggregates, which typically demonstrated lower AP content (Figure 4a,b).

The content of AN in the soil exhibited a declining trend with increasing salinity–alkalinity levels (Figure 4c,d). Notably, under all salinity–alkalinity gradients, the AN content was highest in the B treatment, indicating that *B. tropicus* enhances nitrogen transformation and availability. In contrast, the AN content in the M and MB treatments significantly decreased relative to CK, although the MB treatment exhibited an increase in AN content compared to the M treatment alone (Figure 4c,d). Furthermore, large macro-aggregates contained higher AN content than micro-aggregates, with small macro-aggregates and micro-aggregates typically showing lower AN content, particularly under the S3 salt gradient, where AN content was notably reduced (Figure 4c,d).

### 3.4. Effects of Alfalfa Planting and Inoculation with B. tropicus YJ33 on Soil Enzyme Activity in Saline–Alkali Soil

**Figure 5:**
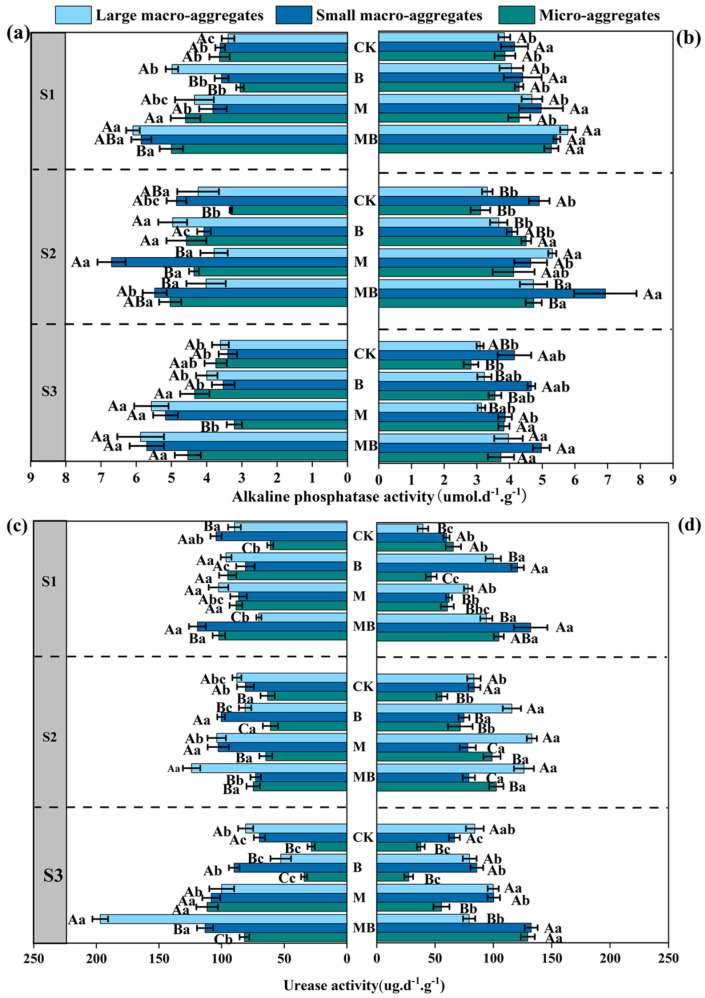
Soil alkaline phosphatase activity (**a**,**b**) and urease activity (**c**,**d**) as affected by different treatments. Measurements for (**a**,**c**) pertain to the 0–10 cm soil layer, while (**b**,**d**) correspond to the 10–20 cm soil layer. Lowercase letters indicated that soil enzyme activity was significantly different among different treatments under the same salinity–alkalinity gradient (*p* < 0.05). Uppercase letters indicated that soil enzyme activity of different particle sizes was significantly different (*p* < 0.05). Values are expressed as mean ± SE (*n* = 4). Abbreviations: S1: low saline soil, S2: medium saline soil; S3: high saline soil, CK: unamended control, B: inoculation of *B. tropicus* YJ33 alone, M: planting of alfalfa alone, and MB: combined alfalfa cultivation with *B. tropicus* YJ33 inoculation.

Both the B and M treatments resulted in a significant increase in S-ALP activity, with the MB treatment demonstrating the highest levels of S-ALP activity, particularly within large and small macro-aggregates (Figure 5a). In comparison to the CK treatment, the B, M, and MB treatments exhibited significantly different levels of enzyme activity, suggesting that the synergistic interaction between alfalfa and microorganisms enhances soil enzyme activity (Figure 5a). This trend was similarly observed in the 10–20 cm soil layer, where the MB treatment continued to show a more pronounced enhancement in S-ALP activity relative to the individual treatments (Figure 5b). Furthermore, S-ALP activity was generally higher in large and small macro-aggregates than in micro-aggregates, indicating that large and small macro-aggregates may offer a more conducive microenvironment for enhancing enzyme activity (Figure 5b).

In the 0–10 cm soil layer, the S-UE activity was significantly enhanced under the MB treatment. The differences among treatments were most pronounced in large and small macro-aggregates, with S-UE activity demonstrating a hierarchical pattern and significant variability across treatment combinations (Figure 5c). In the 10–20 cm soil layer, the MB treatment exhibited higher urease activity compared to the CK, B, and M treatments. Within this soil layer, S-UE activity in large and small macro-aggregates remained significantly higher than in micro-aggregates, with S-UE activity progressively increasing with aggregate size (Figure 5d).

### 3.5. Effects of Planting Alfalfa and Inoculating B. tropicus YJ33 on Composition and Stability of Soil Aggregates in Saline–Alkali Soil

**Figure 6:**
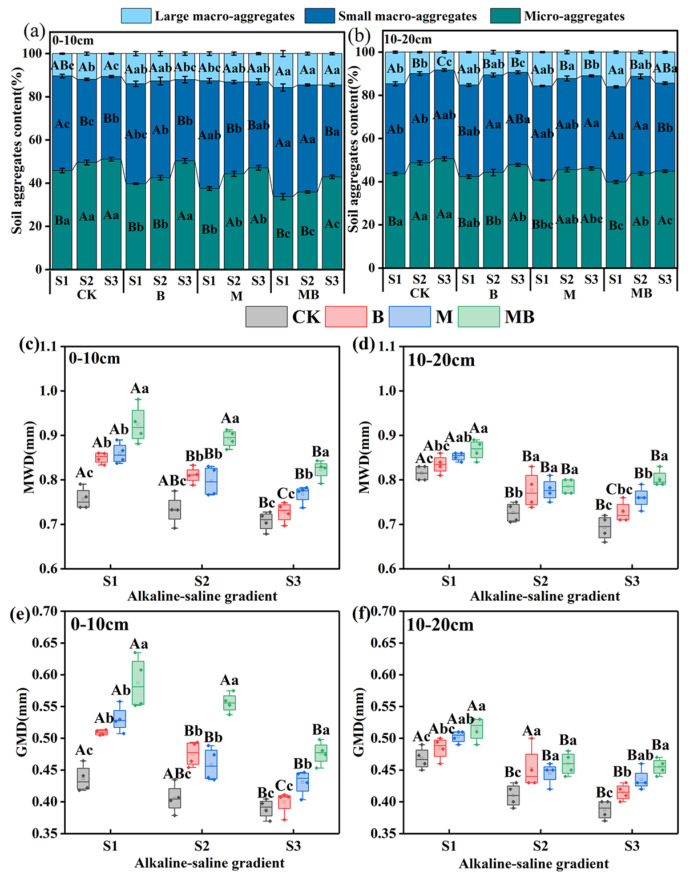
Particle size distribution of soil aggregates in 0–10 cm (**a**) and 10–20 cm (**b**) layers, MWD (**c**,**d**), and GMD (**e**,**f**) as affected by different treatments. Measurements for (**c**,**e**) correspond to the 0–10 cm soil layer, while (**d**,**f**) pertain to the 10–20 cm soil layer. Lowercase letters show significant differences in different treatments within the same salt gradient (*p* < 0.05), and uppercase letters show significant difference in different salt gradients within the same treatment (*p* < 0.05). Values are expressed as mean ± SE (*n* = 4). Abbreviations: MWD: mean weight diameter, GMD: geometric mean diameter, S1: low saline soil, S2: medium saline soil, S3: high saline soil, CK: unamended control, B: inoculation of *B. tropicus* YJ33 alone, M: planting of alfalfa alone, and MB: combined alfalfa cultivation with *B. tropicus* YJ33 inoculation.

This study’s findings indicate that micro-aggregates are predominant in saline–alkaline soils, while large aggregates are the least prevalent. The proportion of micro-aggregates increases with heightened soil salinity–alkalinity, and at the S3 salt gradient, the micro-aggregate content is significantly greater than at the S1 salt gradient (*p* < 0.05) (Figure 6a,b). The B, M, and MB treatments exert a significant influence on the particle size distribution of soil aggregates. Within the same salinity–alkalinity gradient, the MB treatment’s synergistic effect markedly decreases the micro-aggregate content (*p* < 0.05) and significantly enhances the content of large aggregates (*p* < 0.05) (Figure 6a,b). These alterations are consistent across various soil layers.

MWD and geometric GMD exhibit a declining trend with increasing salinity–alkalinity, with MWD and GMD at the S3 salt gradient being significantly lower than those at the S1 salt gradient (Figure 6c–e). Following the B and M treatments, soil MWD and GMD are higher compared to the CK treatment, with the MB treatment demonstrating a superior enhancement effect, exhibiting values significantly higher than CK (*p* < 0.05).

### 3.6. Effects of Soil Physicochemical Properties and Aggregate Stability on Alfalfa Biomass

**Figure 7:**
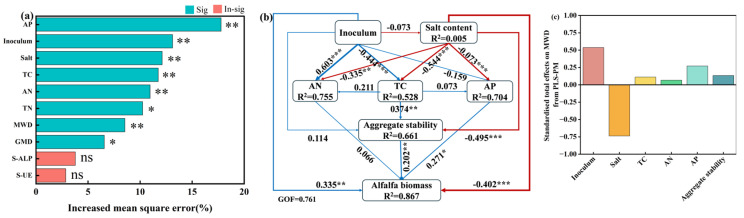
Main factors influencing alfalfa biomass (**a**) and the Partial Least Squares Path Model (PLS-PM) showing the effects of *B. tropicus* inoculation, soil physicochemical properties, and aggregates on alfalfa biomass (**b**), along with the standardized total effects of PLS-PM on alfalfa biomass (**c**). Asterisks indicate significant differences (***, *p* < 0.001; **, *p* < 0.01; and *, *p* < 0.05), and “ns” indicates non-significant differences. In the model, blue lines indicate positive path coefficients, whereas red lines indicate negative path coefficients. The thickness of the arrows corresponds to the magnitude of the standardized path coefficients. The goodness-of-fit (GOF) index is used to assess the model’s fit. Abbreviations: Inoculum: inoculation with *B. tropicus* YJ33, Salt: salt content, TC: total carbon, TN: total nitrogen, AN: available nitrogen, AP: available phosphorus, MWD: mean weight diameter, GMD: mean geometric diameter, S-ALP: soil alkaline phosphatase, and S-UE: soil urease.

A random forest model was employed to predict the potential factors influencing alfalfa biomass (mean square error increase). The analysis revealed that soil AP (17.78%), inoculum (13.12%), salt (12.12%), TC (11.75%), AN (10.96%), TN (10.24%), MWD (8.54%), and GMD (6.56%) significantly affected alfalfa biomass (*p* < 0.05) (Figure 7a). These findings underscore the critical role these factors play in determining alfalfa yield. Conversely, S-ALP activity and S-UE did not exhibit a significant impact on yield, indicating their limited direct influence under the conditions of this study.

Furthermore, a structural equation model (SEM) utilizing the partial least squares (PLS) method elucidated the direct and indirect effects of inoculation, soil nutrients, salinity, and aggregate stability on alfalfa biomass. The model’s goodness of fit (GOF) value was 0.761, suggesting a robust explanation of alfalfa biomass (R^2^ = 0.861). The analysis of the model indicated that inoculation (λ = 0.335, *p* < 0.01), AP (λ = 0.271, *p* < 0.05), and soil aggregate stability (λ = 0.202, *p* < 0.01) exerted significant positive effects on alfalfa biomass. Conversely, salt content (λ = −0.402, *p* < 0.001) demonstrated a significant negative impact on alfalfa biomass (Figure 7b). Additionally, soil salinity exhibited highly significant negative effects on soil TC (λ = −0.544, *p* < 0.001), aggregate stability (λ = −0.495, *p* < 0.001), and AP (λ = −0.682, *p* < 0.001) (Figure 7b). Inoculation was found to have significant positive effects on TC (λ = 0.444, *p* < 0.001) and AN (λ = 0.603, *p* < 0.001) (Figure 7b). Furthermore, soil TC content had a significant positive influence on aggregate stability (λ = 0.374, *p* < 0.01), underscoring the critical role of soil TC in enhancing aggregate stability. Stable aggregates contribute to the formation of soil structure, which subsequently influences alfalfa biomass.

The cumulative effects of inoculation, soil TC, AN, AP, and aggregate stability on alfalfa biomass were found to be positive, whereas the overall effect of salt content was negative. Among these factors, inoculation emerged as the most significant positive contributor (Figure 7c).

## 4. Discussion

### 4.1. Growth-Promoting Effect of B. tropicus YJ33 on Alfalfa Grown in Saline Conditions

As salinity–alkalinity levels increased, there was a reduction in alfalfa plant height, aboveground biomass, and underground biomass. This outcome aligns with the findings of Berna et al. (2024), who reported that long-term high-salt stress caused an over 80% reduction in alfalfa plant height, leaf number, and biomass [37]. Under salt stress, alfalfa exhibited increased root elongation, which contradicts the findings of Lei et al. (2018) that salt stress inhibits root development [38]. This paradox reflects a typical adaptive strategy of alfalfa to saline conditions. Plants subjected to saline–alkaline stress often enhance root growth to acclimate to environmental challenges. Despite this compensatory root development, overall plant growth and biomass accumulation remained constrained [39]. Inoculation with *B. tropicus* YJ33 has been shown to significantly enhance alfalfa plant height, aboveground biomass, root length, and underground biomass, suggesting a beneficial interaction between the microorganism and the plant. This result aligns with the findings of Chen et al. (2024), who reported that *B. tropicus* YJ33 inoculation markedly increased alfalfa biomass, plant height, and root length [36]. Previous research has established that *B. tropicus* possesses phosphorus-solubilizing and nitrogen-fixing capabilities [40], which facilitate the dissolution of insoluble soil nutrients and improve the efficiency of plant nutrient uptake. Additionally, studies have reported that inoculation with *Bacillus cereus* can stimulate root and stem growth, as well as increase the fresh and dry weight of these plant parts. This effect is likely attributable to the production of indole-3-acetic acid (IAA) by the microorganisms, which promotes root proliferation and stimulates cell elongation and division [41]. Consequently, this enhances water and nutrient absorption, leading to increased plant height and overall biomass. Furthermore, inoculation with *B. tropicus* may mitigate the adverse effects of salinity on alfalfa’s physiological processes. Similar investigations into plant–microbe interactions have demonstrated their role in maintaining osmotic balance within plant cells and reducing oxidative damage induced by salt stress [42,43,44].

### 4.2. Improvement of Nutritional Quality of Alfalfa in Saline Soil by B. tropicus YJ33

As salinity and alkalinity levels increase, there is a noted decline in total phosphorus and crude protein content in alfalfa, while the levels of NDF and ADF rise. This trend is likely due to the physiological metabolic stress induced by high-salinity conditions, which impairs phosphorus absorption and utilization, as well as protein synthesis [45]. The observed increase in NDF and ADF content contradicts previous findings [46]. However, the inoculation of alfalfa with *B. tropicus* has been shown to enhance total phosphorus and crude protein content while simultaneously reducing NDF and ADF levels. This suggests a beneficial symbiotic interaction between *B. tropicus* and alfalfa in saline–alkaline environments. Previous studies have indicated that certain beneficial microorganisms secrete organic acids to mobilize phosphorus in the soil, thereby enhancing its availability for plant uptake [47], which aligns with the observed increase in total phosphorus content in this study. Furthermore, related research has demonstrated that inoculation with *Bacillus subtilis* decreases fiber content and increases crude protein content [48]. While this study primarily focused on corn silage, the impact of Bacillus on fiber content is noteworthy. The enhanced effects of inoculating with *B. tropicus* suggest a promising biological amelioration strategy for alfalfa cultivation in saline–alkaline soils, potentially improving both yield and quality. This approach could further facilitate the agricultural development and utilization of saline–alkaline lands.

### 4.3. The Effects of Alfalfa and B. tropicus YJ33 on Soil Nutrients

Soil aggregates, as critical components of soil structure, significantly influence the storage and availability of soil nutrients. This study revealed that treatments B, M, and MB led to increased levels of TC, TN, and AP in large aggregates. This result is similar to the research result of Tian et al. [25]. Li et al. [49] showed a significant positive correlation between the abundance of *Bacillus* and the levels of TN and AP in soil. This differs from the findings presented here. The observed phenomena may be attributed to the metabolic activities of *Bacillus*, which facilitate nutrient transformation and availability in the soil [50]. The research of Xiao et al. [51] found that large aggregates can store more TC and TN, which is closely related to our research. It might be because large aggregates (such as >2 mm) have a relatively large pore structure. These pores can physically protect organic carbon and nitrogen compounds. For instance, organic matter may be adsorbed onto the surfaces of large particles or reside within the pores between large particles, thereby reducing microbial decomposition [52]. Nonetheless, this study also revealed a significant decrease in AN content in the soil following M and MB treatments, potentially due to plant uptake and utilization of AN. It is also possible that the treatment of M and MB reduces the proportion of small aggregates, and small aggregates (such as <0.25 mm) contribute more to the available nitrogen [53].

### 4.4. The Effects of Alfalfa and B. tropicus YJ33 on Enzyme Activity of Soil

S-ALP is implicated in phosphorus acquisition in the soil and plays a vital role in the phosphorus cycle within the soil ecosystem [54]. It enhances phosphorus availability in the soil, thereby supplying adequate phosphorus nutrients for plant growth and promoting root development. Furthermore, plant roots can improve soil aggregate stability through compression and the infiltration of organic matter [55]. In this study, the MB treatment exhibited elevated S-ALP activity. Huang et al. (2023) investigated that co-inoculation of *Aspergillus mycorrhizal fungi* (AMF) with *phosphate-solubilizing bacteria* (PSB) significantly increased S-ALP activity and promoted organic phosphorus mineralization [56]. Liu (2020) found that AMF expanded root uptake through mycelial network while PSB secreted organic acids to solubilize refractory phosphorus, and they synergistically enhanced phosphorus effectiveness [57]. MB treatment may enhance microbial community functioning through a similar mechanism, and increased microbial populations in the soil may lead to an increased demand for phosphorus [58]. Notably, alkaline phosphatase activity was greater in large aggregates compared to micro-aggregates. This is consistent with the results of the tillage study by Bian et al. (2025) [59]. It may be related to the fact that larger agglomerates contain more plant debris and less humic organic matter [60]. S-UE, an enzyme associated with nitrogen acquisition in soil, facilitates nitrogen mineralization, and variations in TN content can affect urease activity [61]. The MB treatment enhanced urease production and accumulation, with urease activity being more pronounced in large aggregates than in small aggregates. Richardson et al. (2011) proposed that microorganisms accelerate urea hydrolysis and promote nitrogen mineralization by secreting urease, while the pore structure of macro-aggregates may facilitate gas diffusion (e.g., reducing anaerobic inhibition), thereby enhancing enzyme activity [62].

### 4.5. Improvement of Stability of Soil Aggregates in Saline Soil by Alfalfa and B. tropicus YJ33

The MWD and GMD of soil aggregates are widely recognized as key indicators for assessing the particle size distribution and water stability characteristics of soil aggregates. These metrics are employed to quantitatively evaluate soil aggregate stability, with higher values signifying enhanced stability. In this study, it was observed that the B, M, and MB treatments resulted in a reduction in the content of micro-aggregates and an increase in the content of large aggregates, thereby leading to an increase in soil MWD and GMD.

These alterations indicate a marked improvement in soil structure, aligning with findings from previous research. Numerous studies have identified the composition and stability of soil aggregates as critical indicators of soil quality. The observed increase in the content of large aggregates, along with the rise in MWD and GMD, suggests that soil particles are more cohesively and stably bound, thereby enhancing soil aeration, permeability, and water retention [63].

This structural enhancement fosters a more conducive environment for soil microbial activity, thereby facilitating nutrient cycling and transformation within the soil [64]. As a leguminous species, alfalfa contributes significantly to soil aggregate formation through its root exudates and residues [65]. Specifically, polysaccharides and proteins function as adhesives for soil particles, aiding in the transformation of small aggregates into larger ones. Inoculation with *B. tropicus* can affect soil aggregate properties in multiple ways. Firstly, the bacteria may produce extracellular polysaccharides and other compounds that directly contribute to the formation and stabilization of soil aggregates [66]. Secondly, it may establish a symbiotic relationship with alfalfa roots, enhancing root attachment to soil particles by modifying the soil microbial community structure, thereby indirectly influencing soil aggregate formation and stability [67].

### 4.6. The Effects of Inoculation with B. tropicus YJ33 on Soil Aggregates and Alfalfa Growth

The predominant influence of AP on alfalfa yield aligns with prior research, underscoring phosphorus as a critical nutrient in promoting plant growth and biomass production [68]. In leguminous species like alfalfa, phosphorus is vital for energy transfer and root development. Similarly, TN is essential for alfalfa, with evidence indicating that nitrogen significantly enhances feed quality and yield, particularly under nutrient-limited conditions [69]. Soil salinity also plays a crucial role, with studies demonstrating that high salinity adversely affects alfalfa germination, root elongation, and overall growth [70]. However, moderate salinity levels may interact with other nutrients, resulting in diverse effects on plant performance across different environments. Notably, the impact of soil enzyme activities (such as S-UE and S-ALP) was found to be insignificant, suggesting that these factors may exert an indirect or secondary influence on alfalfa biomass. Related studies have indicated that in intensive cropping systems, the direct effects of soil structure and microorganisms on yield may be obscured by nutrient availability [71].

Salinity exerts a substantial negative influence on soil TC, AN, and AP, which not only directly inhibits plant growth but also amplifies its detrimental impact on plant yield by diminishing nutrient availability. Soil TC significantly enhances soil aggregate stability, underscoring its pivotal role in maintaining soil structure [72]. Stable soil aggregates improve soil permeability, aeration, and nutrient availability, thereby indirectly supporting alfalfa biomass [73]. The enhancement of soil aggregate stability contributes to a robust soil structure conducive to root development and nutrient cycling. Although the direct effect of soil aggregate stability on biomass is limited, its intermediary role in enhancing nutrient retention and mitigating the adverse effects of salinity is of critical importance.

## 5. Conclusions

This study demonstrates that the synergistic interaction between *B. tropicus* YJ33 and alfalfa effectively mitigates soil salinization challenges by improving aggregate stability and nutrient availability in saline–alkali soils. The combined application of microbial inoculation and plant cultivation significantly enhanced soil structure and nutrient cycling, which directly translated into improved alfalfa biomass and nutritional quality. These findings highlight the potential of *B. tropicus* YJ33 as a bioaugmentation agent for sustainable saline soil remediation, offering a dual benefit of soil health restoration and crop productivity enhancement. Future research should focus on field-scale validation of this plant-microbe synergy, optimization of inoculation protocols under varying salinity gradients, and exploration of the molecular mechanisms driving microbial-mediated aggregate formation. Additionally, extending this approach to other salt-tolerant crops and integrating multi-omics analyses could unlock broader applications in global saline land rehabilitation and climate-resilient agriculture.

## Data Availability

The original data generated in this study are included in this article. Further enquiries can be directed to the corresponding authors.
